# Evolution of multi-parametric MRI quantitative parameters following transrectal ultrasound-guided biopsy of the prostate

**DOI:** 10.1038/pcan.2015.33

**Published:** 2015-07-21

**Authors:** A Latifoltojar, N Dikaios, A Ridout, C Moore, R Illing, A Kirkham, S Taylor, S Halligan, D Atkinson, C Allen, M Emberton, S Punwani

**Affiliations:** 1 Centre for Medical Imaging, University College London, London, UK; 2 Department of Urology, University College London Hospital, London, UK; 3 Department of Radiology, University College London Hospital, London, UK

## Abstract

**Background:**

To determine the evolution of prostatic multi-parametric magnetic resonance imaging (mp-MRI) signal following transrectal ultrasound (TRUS)-guided biopsy.

**Methods:**

Local ethical permission and informed written consent was obtained from all the participants (*n*=14, aged 43–69, mean 64 years). Patients with a clinical suspicion of prostate cancer (PSA range 2.2–11.7, mean 6.2) and a negative (PIRAD 1–2/5) pre-biopsy mp-MRI (pre-contrast T1, T2, diffusion-weighted and dynamic-contrast-enhanced MRI) who underwent 10-core TRUS-guided biopsy were recruited for additional mp-MRI examinations performed at 1, 2 and 6 months post biopsy. We quantified mp-MRI peripheral zone (PZ) and transition zone (TZ) normalized T2 signal intensity (nT2-SI); T1 relaxation time (T_10_); diffusion-weighted MRI, apparent diffusion coefficient (ADC); dynamic contrast-enhanced MRI, maximum enhancement (ME); slope of enhancement (SoE) and area-under-the-contrast-enhancement-curve at 120 s (AUC_120_). Significant changes in mp-MRI parameters were identified by analysis of variance with Dunnett's post testing.

**Results:**

Diffuse signal changes were observed post-biopsy throughout the PZ. No significant signal change occurred following biopsy within the TZ. Left and right PZ mean nT2-SI (left PZ: 5.73, 5.16, 4.90 and 5.12; right PZ: 5.80, 5.10, 4.84 and 5.05 at pre-biopsy, 1, 2 and 6 months post biopsy, respectively) and mean T_10_ (left PZ: 1.02, 0.67, 0.78, 0.85; right PZ: 1.29, 0.64, 0.78, 0.87 at pre-biopsy, 1, 2 and 6 months post biopsy, respectively) were reduced significantly (*P*<0.05) from pre-biopsy values for up to 6 months post biopsy. Significant changes (*P*<0.05) of PZ-ME and AUC_120_ were observed at 1 month but resolved by 2 months post biopsy. PZ ADC did not change significantly following biopsy (*P*=0.23–1.0). There was no significant change of any TZ mp-MRI parameter at any time point following biopsy (*P*=0.1–1.0).

**Conclusions:**

Significant PZ (but not TZ) T2 signal changes persist up to 6 months post biopsy, whereas PZ and TZ ADC is not significantly altered as early as 1 month post biopsy. Caution must be exercised when interpreting T1- and T2-weighted imaging early post biopsy, whereas ADC images are more likely to maintain clinical efficacy.

## Introduction

Tumour localization using magnetic resonance imaging (MRI) is increasingly important for the management of prostate cancer. Pelvic imaging using T1- and T2-weighted MRI has conventionally been used to locally stage prostate cancer following histological confirmation by transrectal ultrasound (TRUS)-guided biopsy.

However, biopsy itself can hamper localization of tumour on anatomical MRI. For example, prostate cancer within the peripheral zone (PZ) is typically of low T2-weighted signal intensity compared with surrounding normal tissue. Consequently, reduction of T2-weighted signal intensity from normal tissue observed after biopsy^1^ can mask significant cancer or cause overestimation of disease extent.^2^

Previous work suggests that TRUS biopsy-induced anatomical MRI signal changes can persist for up to 8 weeks^3^ and that resolution is unpredictable.^1, 4^ A strategy of delaying MRI following biopsy has therefore evolved, with recommendations, based on available evidence, postponing staging MRI until at least 3 weeks^4^ and up to 8 weeks after biopsy.^3^

More recently, MRI has emerged as a tool to detect prostate cancer in patients with elevated risk. State-of-the-art multi-parametric MRI (mp-MRI; T2-weighted, diffusion and dynamic contrast-enhanced imaging) performed before biopsy enables biopsy to be targeted, which may be more effective to diagnose clinically significant disease.^5, 6, 7^ Indeed, the recent update of National Institute for Health and Care Excellence guidelines on prostate cancer management recommend that mp-MRI is used for patients with negative 10–12 cores TRUS biopsy (http://www.nice.org.uk/guidance/cg175/chapter/recommendations). Understanding the extent and duration of mp-MRI changes precipitated by biopsy is central to minimizing their misinterpretation and to identify the most appropriate delay between biopsy and subsequent mp-MRI. However, work assessing the evolution of imaging features following biopsy is limited and, where present, such work has focused on qualitative assessment and compared pre-biopsy mp-MRI with a single, but variable interval post-biopsy mp-MRI. The natural history of mp-MRI signal change following biopsy has not been addressed.^1, 2, 3, 4^

This study systematically and quantitatively describes mp-MRI signal evolution over a period of 6 months following TRUS-guided biopsy.

## Materials and methods

Permission was obtained from the institutional ethics committee and informed written consent was obtained from all the participants (REC number: 08/H0714/21).

### Patient recruitment

Fourteen patients (aged 43–69, mean 64 years) with (i) an elevated PSA (range 2.2–11.7, mean 6.2) and (ii) a negative pre-biopsy prostate mp-MRI report (PI-RADS score of equal or less than 2/5)^8^ were recruited prospectively. Prostate volume calculated from pre-biopsy T2-weighted images ranged from 17 to 80 cm^3^ (mean 52.7 cm^3^). All the patients underwent a standard ultrasound-guided 10-core TRUS biopsy procedure.^9^ The patients then returned for additional mp-MRI scans performed at 1, 2 and 6 months post biopsy.

### Multi-parametric MRI protocol

Participants were imaged using a 1.5 T MRI scanner (Avanto; Siemens, Erlangen, Germany). With the patient supine, the manufacturers body coil was used for signal excitation and a six-element surface phased-array coil used for reception. To reduce peristalsis, 20 mg of butylscopolamine bromide (Buscopan; Boehringer-Ingelheim, Ingelheim, Germany) was administered intravenously before image acquisition.

Three-millimetre-thick axial and coronal small field of view T2-weighted images were acquired using a turbo spin echo sequence. Five-millimetre-thick diffusion-weighted imaging was performed using short tau inversion recovery echo planar imaging at b0, 150, 500 and 1000 s mm^−^^2^ (each averaged 16 times). Apparent diffusion coefficient (ADC) maps were generated using all the acquired b-values. For T1 quantification, axial pre-contrast T1-weighted images at multiple flip angles were acquired using a volume-interpolated gradient echo sequence. Dynamic contrast-enhanced (DCE) imaging was performed during free breathing using repeated volume-interpolated gradient echo acquisitions at a fixed flip angle with a temporal resolution of 17 s and a total of 35 measures. A total 0.1 mmol kg^−1^ gadoterate meglumine (Dotarem, Guerbet, Villepinte, France) followed by a 10 ml saline flush was injected intravenously at 3 ml s^−1^ at the start of the sixth measure. Full sequence parameters are provided in Table 1.

### Image analysis

Image analysis was performed using Jim software (Jim, Xinapse Systems, Version 6, Leicester, UK) by two observers in consensus, with 10 and 3 years experience of interpretation of prostate MRI, respectively. Pre-contrast multiple flip angle T1-weighted, axial T2-weighted, ADC maps and DCE images were evaluated for each patient at each time point.

To provide patient level data, the prostate was manually segmented by individually contouring the left and right PZ and left and right transition zone (TZ) on each axial slice depicting the prostate at each time point.

To assess cohort level changes, the mean value from whole left and right peripheral zone, and whole left and right TZ (that is, across all the slices of a patient) on pre-contrast T1-weighted, T2-weighted and ADC images was derived for each patient at each time point.

Furthermore, a single region of interest was placed centrally within the right obturator internus muscle on T2-weighted images of each patient to act as a reference for normalization of T2-weighted signal. Normalized T2-weighted signal intensities (nT2-SI) were calculated by deriving a ratio of prostate zone: obturator internus signal.

Curve fitting of signal intensity measurements at multiple flip angles was used to derive T1 relaxation time (T_10_). For dynamic contrast-enhanced images, a single signal intensity time curve was derived from the mean of all voxels within each zone. Initial slope of enhancement (SoE) and maximum enhancement (ME) was extracted as previously reported.^10^ In addition, the area under the contrast-enhancement time curve from arrival of contrast within the prostate to 120 s (AUC_120_) was determined.

### Statistical analysis

A repeated measures analysis of variance followed by Dunnett's multiple comparison post testing; and a ordinary one-way analysis of variance followed by Dunnett's multiple comparison post testing was used to identify significant differences between mean mp-MRI parameters for each post-biopsy time point compared with pre-biopsy baseline values on a cohort level and per-patient level, respectively.

## Results

All the patients attended each of the four mp-MRI scanning sessions. All pre-biopsy mp-MRI studies were prospectively reported negative for significant cancer (PI-RADS 1–2/5). In addition, even with biopsy results available, in retrospect, no tumour was localized on pre-biopsy MRI studies. There was no significant biopsy complication. Five patients were diagnosed with 1 core of 1mm Gleason 3+3 tumour and one patient had 1 core of 3mm of Gleason 3+4. All cores from the remaining patients were benign. No treatment was administered to biopsy-positive patients during the follow-up period. Mean values for all quantitative parameters pre- and post-biopsy time points are given in Table 2.

In all the patients, diffuse signal changes were evident throughout the PZ following biopsy, whereas no perceptible change was evident within the TZ.

### Normalized T2-weighted signal intensity

**Figure 1:**
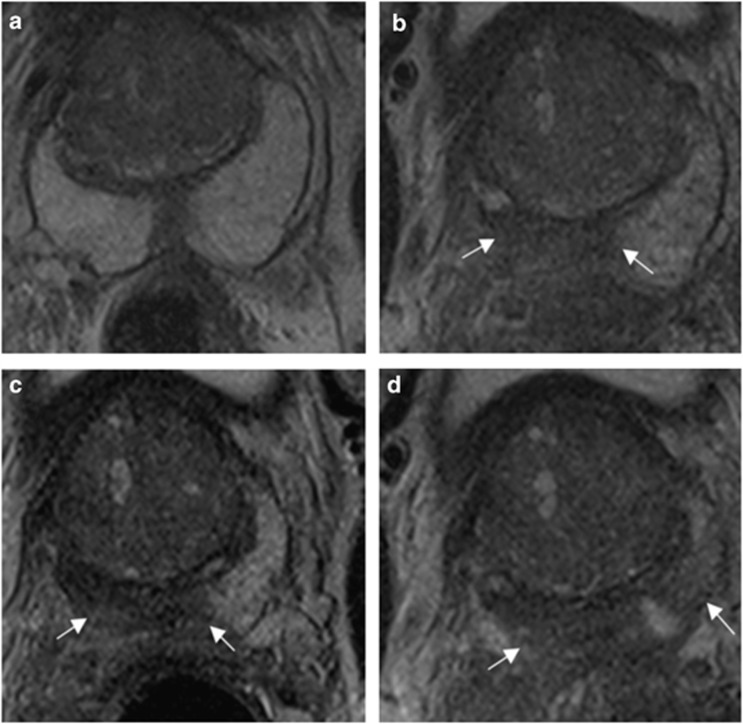
Representative axial T2-weighted images at the mid-gland demonstrate (**a**) normal peripheral zone T2 signal intensity pre-biopsy; and reduction (white arrows) in the peripheral zone T2 signal intensity at (**b**) 1 month, (**c**) 2 months and (**d**) 6 months post biopsy.

**Figure 2:**
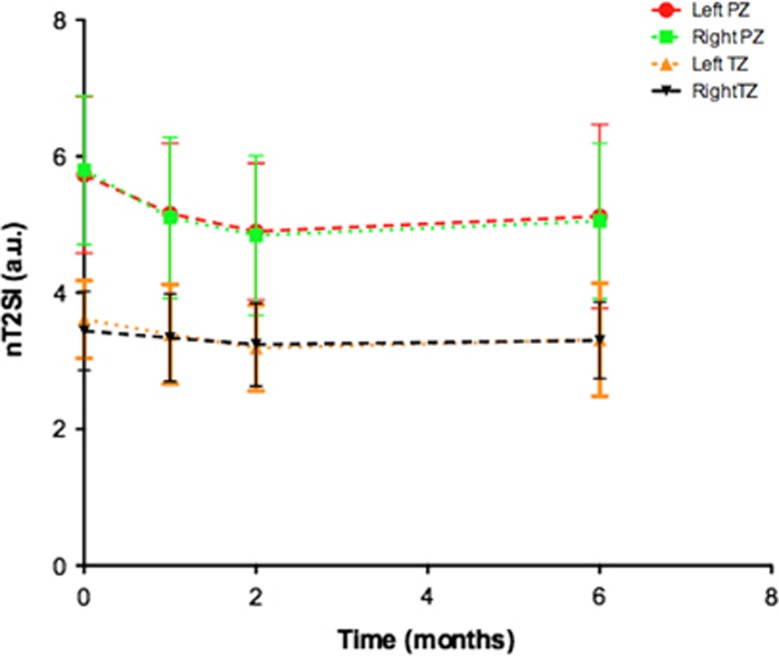
Temporal change of mean (error bars±s.d.) normalized T2 signal intensity (nT2 SI) in the (**a**) left peripheral zone (PZ; red line), (**b**) right peripheral zone (green line), (**c**) left transition zone (TZ; orange line) and (**d**) right transition zone (black line). Significant changes are indicated in Table 2.

A typical example of T2 signal evolution is shown in Figure 1. Two patients were excluded from the analysis of nT2-SI change because baseline T2-weighted MRI imaging parameters violated the required trial protocol defined in Table 1. Temporal change of mean pre-biopsy nT2-SI (*n*=12) for left and right peripheral and transition zone is illustrated in Figure 2. For both left and right peripheral zones, nT2-SI was reduced significantly compared with pre-biopsy values at 1, 2 and 6 months (left PZ mean nT2-SI; 5.73, 5.16, 4.90 and 5.12 at pre-biopsy, 1, 2 and 6 months post-biopsy, respectively, *P*<0.05; right PZ mean nT2-SI; 5.80, 5.10, 4.84 and 5.05 at pre-biopsy, 1, 2 and 6 months post-biopsy, respectively, *P*<0.05). There was no significant difference between transition zone nT2-SI between baseline and any post-biopsy time point (*P*=0.10 to 0.82; Table 2). Patient level PZ and TZ nT2-SI change following TRUS biopsy are presented in Table 3 (a) and (b), respectively. Significant change from baseline of nT2-SI was evident in the PZ in 12/12 patients, and in the TZ in 9/12 patients at one or more post-biopsy time points.

### Diffusion-weighted imaging—apparent diffusion coefficient

**Figure 3:**
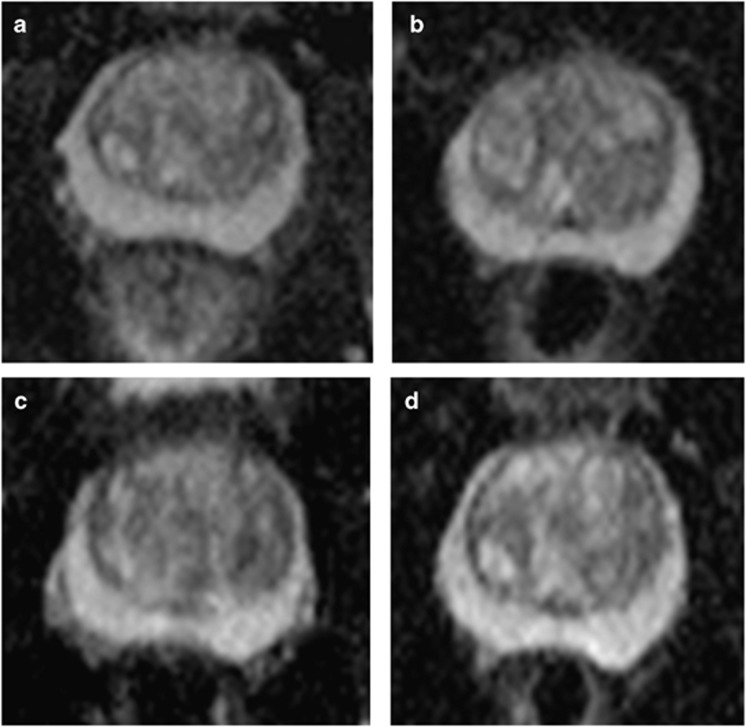
Representative axial apparent diffusion coefficient (ADC) maps at the mid-gland level from a patient within the cohort demonstrate (**a**) normal peripheral zone ADC pre-biopsy; and stable ADC at (**b**) 1 month, (**c**) 2 months and (**d**) 6 months post biopsy.

**Figure 4:**
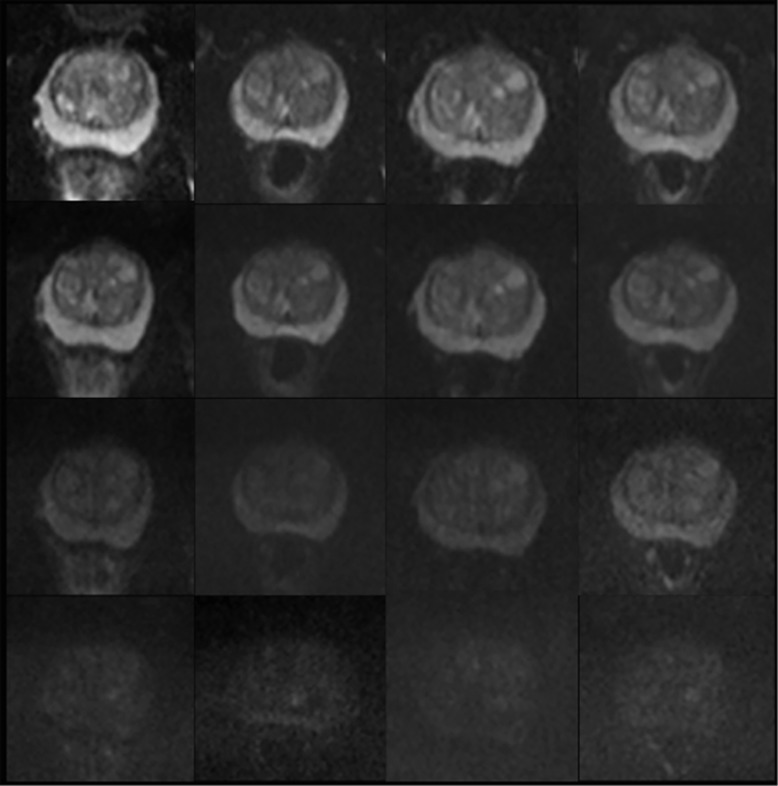
Representative axial multiple b-value diffusion-weighted images at the mid-gland level for the apparent diffusion coefficient maps illustrated in Figure 3. Columns (left to right) represent baseline, 1-, 2-, 6-month post-biopsy time points. Rows (top to bottom) represent b0, b150, b500 and b1000 images. No visual change is evident from baseline for any diffusion weighting at 1, 2 and 6 months post biopsy.

**Figure 5:**
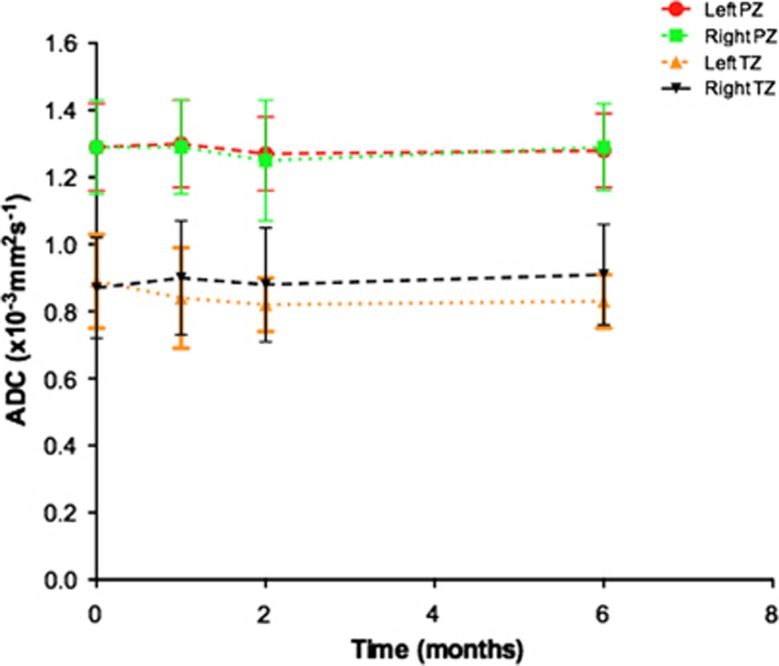
Temporal changes of mean apparent diffusion coefficient (ADC; error bars±s.d.) in the (**a**) left peripheral zone (PZ; red line), (**b**) right peripheral zone (green line), (**c**) left transition zone (TZ; orange line) and (**d**) right transition zone (black line). No significant changes were observed in any zone (Table 2).

An example of temporal ADC and source b-value diffusion-weighted images changes are illustrated in Figures 3 and 4, respectively. Mean pre-biopsy ADC (*n*=14) for left and right peripheral and transition zone is shown in Table 2 and Figure 5. There was no significant difference in baseline ADC for any zone when compared with post-biopsy time points (*P*=0.23 to 1.0). Patient level PZ and TZ ADC change following TRUS biopsy are presented in Table 3 (a) and (b), respectively. Significant change from baseline of ADC was evident in the PZ in 7/14 patients, and in the TZ in 7/14 patients at one or more post-biopsy time points.

### Pre-contrast T1-weighted MRI

**Figure 6:**
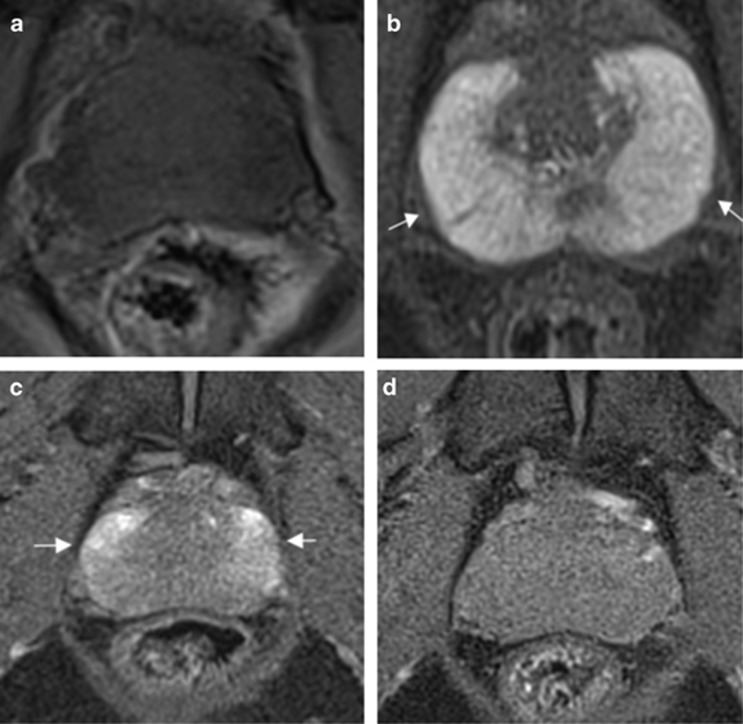
Representative axial T1-weighted images at the mid-gland level demonstrate (**a**) normal peripheral zone T1 signal intensity pre-biopsy; and increase (white arrows) in the peripheral zone T1 signal intensity at (**b**) 1 month, (**c**) 2 months and (**d**) 6 months post biopsy.

**Figure 7:**
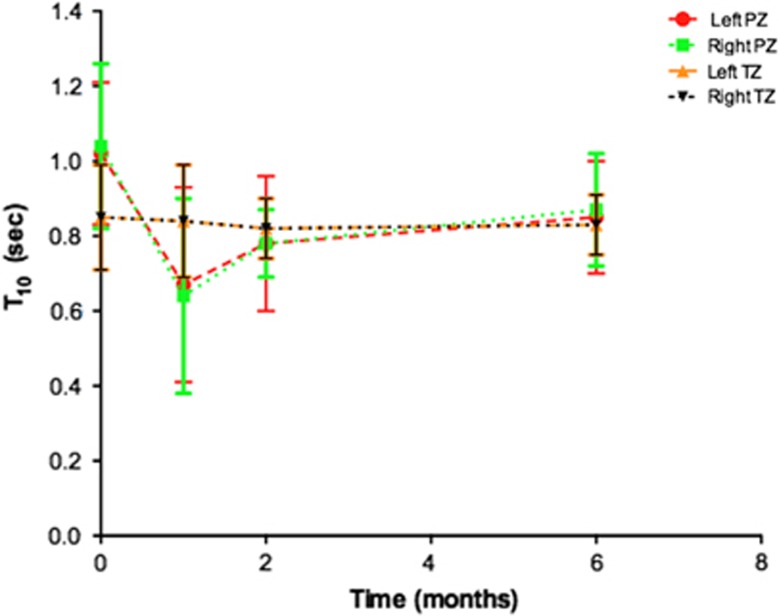
Temporal changes of mean T1 relaxation rate (T_10_; error bars±s.d.) in the (**a**) left peripheral zone (PZ; red line), (**b**) right peripheral zone (green line), (**c**) left transition zone (TZ; orange line) and (**d**) right transition zone (black line). Significant changes are indicated in Table 2.

Evolution of T1 signal is illustrated in Figure 6. Mean pre-biopsy T_10_ (*n*=14) for left and right peripheral and transition zone is depicted in Table 2. T_10_ was significantly lower at 1 month, 2 months and 6 months for the left peripheral zone (mean T_10_; 1.02, 0.67, 0.78, 0.85 at pre-biopsy, 1, 2 and 6 months post biopsy, respectively, *P*<0.05; Figure 7) and at 1 month and 2 months for the right peripheral zone (mean T_10_; 1.29, 0.64, 0.78, 0.87 at pre-biopsy, 1, 2 and 6 months post biopsy, respectively, *P*<0.05; Figure 7). There was no significant difference between pre-biopsy T_10_ and 1 month, 2 months and 6 months T_10_ within left and right transition zones (*P*=0.76 to 1.0; Figure 7). Patient level PZ and TZ T_10_ change following TRUS biopsy are presented in Table 3 (a) and (b), respectively. Significant change from the baseline of T_10_ was evident in the PZ in 12/14 patients and in the TZ in 4/14 patients at one or more post-biopsy time points.

### Dynamic contrast-enhanced imaging

There was no consistency between changes on both sides of the gland for DCE MRI parameters. Mean ME (*n*=14) at 1 month post biopsy was significantly lower within the right peripheral zone (right PZ mean ME; 1.01 and 0.76 at pre-biopsy and 1 month post biopsy, respectively, *P*<0.05); and mean AUC_120_ (*n*=14) was significantly higher at 1 month post biopsy (*P*<0.05) within the left transition zone compared with pre-biopsy values (left TZ mean AUC_120_; 2.12 and 3.39 at pre-biopsy and 1 month post biopsy, respectively). There was no other significant difference for ME, SoE and AUC_120_ between pre-biopsy and post-biopsy time points (*P*=0.06 to 1.0).

## Discussion

Our study documents the natural history of biopsy-induced mp-MRI signal changes and their effect on derived quantitative mp-MRI parameters. We observed significant changes in nT2-SI and T_10_ following biopsy persisting up to 6 months following biopsy. We found no significant change for ADC when pre-biopsy values were compared with any post-biopsy time point. For dynamic contrast-enhanced MRI-derived parameters, we found significant but inconsistent changes.

Our work confirms that, as observed in the works of others, peripheral zone T2-weighted signal intensity is reduced significantly 1 month following TRUS biopsy.^11^ Moreover, we found that a small (mean 12%) but significant reduction in T2 signal persists even at 6 months afterwards. Furthermore, we confirmed that nT2-SI within the transition zone does not change significantly following TRUS biopsy. One possible explanation is that the absence of significant change within the transition zone likely reflects undersampling of the anterior gland by the biopsy procedure.^12^ Previous work illustrates that the T2 signal of PZ tumour is on average reduced by 25% compared with normal PZ and TZ.^13^ We demonstrated maximum average change of 15–17% in T2 signal of normal PZ at 2 months post biopsy and 11–13% at 6 months post biopsy. It is possible that persistent changes of T2 signal intensity even after 6 months could potentially hinder MRI diagnostic performance, especially for low-grade and/or diffuse tumour.

Although studies have described changes in T1 signal intensity within the prostate following biopsy,^14^ quantitative changes in T1 relaxation time have not been described previously. We found that T_10_ decreased significantly after biopsy, consistent with the increase in T1 signal intensity reported previously and ascribed to post-biopsy haemorrhage.^14, 15^ In keeping with the evolution of T2 signal changes, T1 signal changes also start to normalize by 6 months post biopsy and remain significantly different compared with pre-biopsy values.

We observed that ADC was not significantly different from pre-biopsy values at 1, 2 and 6 months post biopsy. Rosenkrantz *et al.*^16^ previously compared ADC of normal benign, haemorrhagic peripheral zone and peripheral zone prostate cancer, and found that ADC was reduced in the areas of haemorrhage. They did not report the temporal evolution of ADC change, and patients recruited were imaged at a wide range of intervals following biopsy but grouped for analysis (range 10 to 241 days, mean 63 days). In contrast, our biopsy cohort mp-MRI scans were acquired at pre-specified time points and did not demonstrate any significant change following biopsy at our earliest interval of 1 month. However, we acknowledge that ADC changes have been associated with haemorrhage in neuroimaging, with hyperacute, acute and early subacute stages causing reduced ADC, which then normalize at subacute and chronic stages.^17^ It is possible that Rosenkrantz *et al.*^16^ included post-biopsy patients with acute haemorrhage, while our earliest post-biopsy imaging mp-MRI corresponds to the subacute/chronic stage. Combined with our study, results suggest that ADC change does occur but normalizes in 1 month.

It is difficult to draw firm conclusions from our DCE results. We hypothesized that TRUS biopsy should affect both sides of the gland equally and any resulting spatial differences would average out across patients. We believe that inconsistent significant findings between left and right zones, despite the presence of diffuse PZ signal increase on pre-contrast T1-weighted images, reflect poor repeatability/reproducibility (also evidenced by the relatively large standard deviation of these measurements) of DCE-derived quantitative measures.^18^

We observed diffuse signal changes in all the patients following biopsy. We believe that the volumetric sampling accurately reflects quantitative mp-MRI parameter changes within our population. Moreover, previous work has shown that volumetric analysis of quantitative derived parameters is more repeatable than single-slice region of interest-based quantification.^19^

Our results suggest that if mp-MRI is performed at least 1 month following biopsy, then ADC is least affected and, therefore, will likely be the most reliable method for localizing disease. Also, based on the finding that anterior gland nT2-SI, T_10_ and ADC are not affected significantly even at 1 month, reliable imaging of the anterior gland with mp-MRI following TRUS biopsy is possible and could be performed early in patients with a negative TRUS biopsy and high PSA for rapid assessment of anterior gland cancers that may be undersampled by TRUS biopsy. We believe that ADC maps at 1 month may help imaging assessment of non-targeted biopsy-positive low-risk prostate cancer to exclude undersampled disease, and to evaluate the suitability of intermediate risk biopsy-positive cancer for potential focal therapy.

Finally, the observation that nT2-SI and T_10_ changes persist at 6 months re-highlights that caution should be applied when relying on anatomical imaging alone for detection and staging prostatic cancer.

There are several limitations to our study. First, our choice of imaging time points was pragmatic, based on clinically useful intervals and patient convenience. Accordingly, we did not assess the immediate effects of biopsy nor whether the signal changes induced by biopsy persisted for longer than 6 months. Second, quantitative DCE MRI parameters revealed inconsistent changes with relatively large standard deviations; whether these findings relate to relatively low temporal resolution of our DCE data set remains unclear. Third, we did not assess the impact of post-biopsy changes on quantitative derived parameters from mp-MRI scans that were positive for tumour.

No visible tumour was evident on mp-MRI for patients included within this study. There is a growing consensus that such disease can be monitored with active surveillance.^20^ However, disease that is visible on mp-MRI is more likely to be of higher risk^21^ and, as such, warrants intervention. Hence, we were unable to recruit patients with mp-MRI visible disease, as a 6-month delay to treatment with high-risk prostate cancer was deemed unethical.

We expect, from the work of others, that reduced levels of citrate in areas of prostate cancer will result in reduced post-biopsy haemorrhage within the tumour region compared with normal surrounding prostate tissue.^1, 22^ Indeed, ADC within tumour may be even less susceptible to biopsy effect than indicated by our results.

We have grouped low-risk MRI-non-visible tumour (*n*=6) patients along with those without cancer (*n*=8), given that the radiological phenotype of both is the same, that is, a negative mp-MRI study. This is in keeping with the growing consensus on non-treatment of ‘insignificant' prostate cancer.^23, 24^

In summary, significant PZ (but not TZ) T2 signal changes persist up to 6 months post biopsy, whereas PZ and TZ ADC is not significantly altered as early as 1 month post biopsy. Caution must be exercised when interpreting T1- and T2-weighted imaging early post biopsy, whereas ADC images are more likely to maintain clinical efficacy.

## Figures and Tables

**Table 1:** MRI sequence parameters

	*T2 TSE*	*T1 GRE (T*_*10*_ *quantification)*	*EPI-DWI*	*T1 GRE (DCE quantification)*
TE/TR (ms)	92/5170	2.5/5.61	96/2100	2.5/5.61
Slice thickness (mm)	3	3	5	3
Spacing between slices (mm)	3.3	NA	5	NA
Number of averages	2	1	16	1
Matrix	256 × 256	192 × 192	172 × 172	192 × 192
Echo train length	17	1	1	1
Flip angle (degree)	180	5, 10, 20, 25	90	15
Field of view (mm)	180	258	340	258

Abbreviations: DCE, dynamic contrast enhancement; DWI, diffusion-weighted imaging; EPI, echo planar imaging; GRE, gradient echo; MRI, magnetic resonance imaging; NA, not available; TE, echo time; TR, repetition time; TSE, turbo spin echo.

**Table 2:** Cohort-based change of quantitative MRI parameters

	*Side*	*Scan*	*Mean nT2-SI (s.d.)*	*Mean T*_*10*_ *(s.d.)*	*Mean ADC (s.d.)*	*Mean ME (s.d.)*	*Mean SoE (s.d.)*	*Mean AUC*_*120*_ *(s.d.)*
Peripheral zone	Left	Pre-biopsy	5.73 (1.15)	1.02 (0.19)	1.29 (0.13)	0.93 (0.27)	1.28 (0.49)	1.93 (0.53)
		1-month	5.16 (1.03)^a^	0.67 (0.26)^a^	1.30 (0.13)	0.72 (0.32)	0.91 (0.41)	1.51 (0.63)
		2-month	4.90 (1.00)^a^	0.78 (0.18)^a^	1.27 (0.11)	0.98 (0.21)	1.14 (0.55)	1.99 (0.42)
		6-month	5.12 (1.35)^a^	0.85 (0.15)^a^	1.28 (0.11)	1.00 (0.24)	1.28 (0.75)	2.08 (0.50)
	Right	Pre-biopsy	5.8 (1.09)	1.04 (0.22)	1.29 (0.14)	1.01 (0.31)	0.96 (0.46)	1.57 (0.59)
		1-month	5.10 (1.18)^a^	0.64 (0.26)^a^	1.29 (0.14)	0.76 (0.33)^a^	1.37 (0.66)	2.07 (0.90)
		2-month	4.84 (1.17)^a^	0.78 (0.09)^a^	1.25 (0.18)	0.93 (0.18)	1.09 (0.62)	1.87 (0.44)
		6-month	5.05 (1.14)^a^	0.87 (0.15)	1.29 (0.13)	0.99 (0.27)	1.26 (0.44)	1.86 (0.57)
								
Transition zone	Left	Pre-biopsy	3.61 (0.57)	0.85 (0.14)	0.89 (0.14)	1.41 (0.32)	1.16 (0.68)	2.12 (0.57)
		1-month	3.39 (0.73)	0.84 (0.15)	0.92 (0.14)	1.33 (0.31)	1.58 (1.08)	3.39 (1.31)^a^
		2-month	3.19 (0.63)	0.82 (0.08)	0.91 (0.14)	1.47 (0.23)	1.62 (1.41)	3.09 (1.32)
		6-month	3.31 (0.73)	0.83 (0.08)	0.94 (0.15)	1.44 (0.20)	1.71 (0.82)	2.68 (0.55)
	Right	Pre-biopsy	3.44 (0.58)	0.87 (0.11)	0.87 (0.15)	1.37 (0.33)	1.32 (0.65)	2.50 (0.83)
		1-month	3.34 (0.64)	0.88 (0.09)	0.9 (0.17)	1.37 (0.20)	1.29 (0.65)	2.52 (0.52)
		2-month	3.24 (0.61)	0.87 (0.08)	0.88 (0.17)	1.47 (0.17)	1.85 (0.86)	2.78 (0.44)
		6-month	3.30 (0.56)	0.84 (0.08)	0.91 (0.15)	1.45 (0.24)	1.34 (0.88)	2.68 (0.58)

Abbreviations: ADC, apparent diffusion coefficient; AUC_120_, area under contrast-enhancement time curve up to 120 s; ME, maximum enhancement of contrast-enhancement time curve; MRI, magnetic resonance imaging; nT2-SI, normalized T2-weighted signal intensity; SoE, slope of enhancement of contrast-enhancement time curve; T_10_, T1 relaxation time.

a Significant change (*P*<0.05) compared with pre-biopsy value.

**Table 3:**
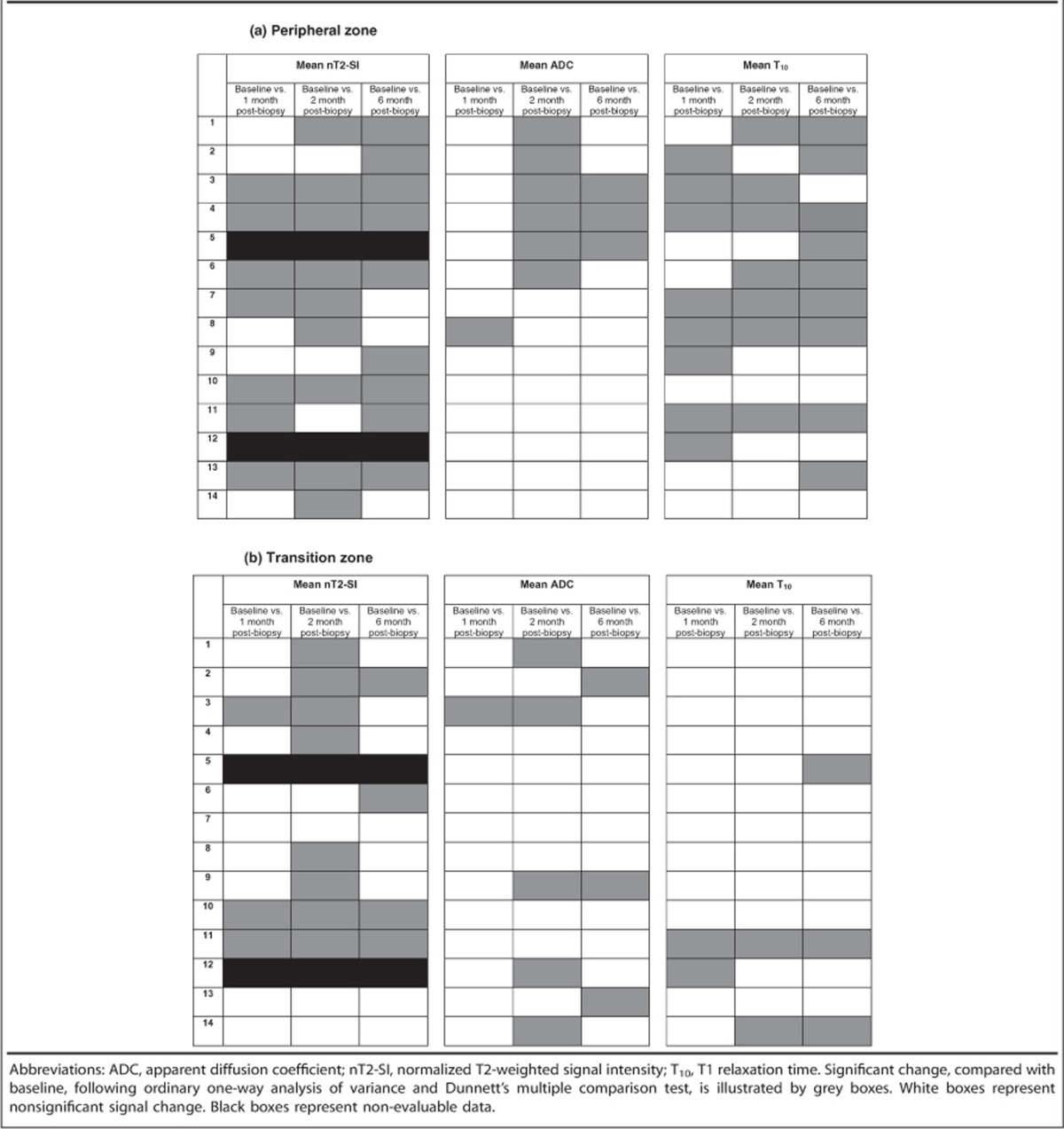
Post-biopsy patient level nT2-SI, ADC and T_10_ signal change
